# Seroprevalence and risk factors of hepatitis B, C and D virus infection amongst patients with features of hepatitis in a referral hospital in Botswana: A cross-sectional study

**DOI:** 10.4102/sajid.v36i1.275

**Published:** 2021-07-19

**Authors:** Sajini Souda, Julius C. Mwita, Francesca Cainelli, Naledi B. Mannathoko, Motswedi Anderson, Sikhulile Moyo

**Affiliations:** 1 Department of Pathology, Faculty of Medicine, University of Botswana, Gaborone, Botswana; 2 Department of Internal Medicine, Faculty of Medicine, University of Botswana, Gaborone, Botswana; 3 Department of Medicine, Raffles Medical Group, Phnom Penh, Cambodia; 4 Department of Biomedical Science, Faculty of Medicine, University of Botswana, Gaborone, Botswana; 5 Botswana Harvard AIDS Institute Partnership, Gaborone, Botswana

**Keywords:** hepatitis B virus infection, hepatitis D virus infection, hepatitis C virus infection, human immunodeficiency virus infection, prevalence, risk factors, liver disease, Botswana

## Abstract

**Background:**

Viral hepatitis is a major global health problem. There is a paucity of data from Botswana on the seroprevalence of markers of hepatitis. The objective of the study was to determine the seroprevalence and risk factors of hepatitis B virus (HBV), hepatitis D virus (HDV) and hepatitis C virus (HCV) infections in patients with clinical features of hepatitis and/or altered liver function tests.

**Method:**

This cross-sectional study was done at Princess Marina Hospital (PMH) in Gaborone, Botswana, from February 2015 to July 2016. It involved 328 adult patients with any of the following: jaundice, history of liver disease and/or increased serum aspartate aminotransferase (AST), alanine aminotransferase (ALT), and serum bilirubin of > 2 times the upper limit of normal (ULN).

**Results:**

Active or chronic active hepatitis (hepatitis B surface antigen [HBsAg] positive) was identified in 46.7% of patients. Antibodies to HDV infection were detected in 4.6% of the HBsAg-positive patients and antibodies to HCV infection in 4.3% of the study patients. Immunity against HBV infection was noted in 34.5% of patients. Human immunodeficiency virus (HIV) co-infection was self-reported by 42.7% of HBsAg-positive patients with known HIV status.

**Conclusion:**

High prevalence rate of HBV, HCV, HDV infection and HIV co-infection was observed in patients with liver disease attending PMH.

## Introduction

Viral hepatitis is a serious public health problem, responsible for an estimated 1.4 million annual deaths globally, largely because of related liver cancer, cirrhosis and in a much lower proportion to acute liver failure.^1^

Hepatitis B virus (HBV) and hepatitis C virus (HCV) contribute to 47% and 48% of these deaths, respectively.^1^ The high burden of viral hepatitis-related morbidity and mortality led to the creation of the Global Health Sector Strategy on Viral Hepatitis 2016–2021, aiming at a 90% reduction of new viral hepatitis infections and a 65% reduction in deaths resulting from viral hepatitis by 2030.^1^

Two billion people are estimated to have evidence of past or present infection with HBV worldwide.^1^ Hepatitis B virus infection has resulted in 887 000 deaths in 2015, mostly from complications related to cirrhosis and hepatocellular carcinoma.^2^ Co-infection of HBV and hepatitis D virus (HDV) occurs in 5% of HBV-infected individuals (approximately 15–20 million people) worldwide and also leads to more severe liver disease.^3^ Studies in Africa have reported the seroprevalence rate of HBV and HDV co-infections ranging from 0% – 0.6% in South Africa to 5% – 58% in Egypt with 8.39% in sub-Saharan Africa.^2,3,4^

Although HCV and HBV share the same routes of transmission, global prevalence of HCV infection is lower (1%, 71 million people) than that of HBV (3.5%) infection.^1^ In Africa, the prevalence of HCV infection is between 0.1% and 17.5%, with 2% – 2.9% in sub-Saharan Africa.^5,6^ HCV causes 399 000 deaths each year from mostly cirrhosis and hepatocellular carcinoma.^1^

The high prevalence of the human immunodeficiency virus (HIV) infection partly contributes to the burden of viral hepatitis in Africa. Globally 2.7 million people (7.4%) living with HIV infection are co-infected with HBV.^1,7^ Of this, 1.96 million (71%) live in the sub Saharan African region (SSA).^8^ Amongst the HIV-infected patients worldwide, HCV/HIV co-infection is reported in 6.2 % (2.3 million).^1^

Studies carried out globally in patients with liver diseases have shown a higher prevalence of HBV, HCV and HDV infections. Prevalence of 21% – 61.4% for HBV, of 1% – 66.7% for HDV and of 6% – 43% for HCV have been reported from different parts of the world amongst patients with liver disease.^3,9,10,11,12,13,14^

In Botswana, most of the published data on the prevalence of HBV and HCV infections are from studies conducted amongst HIV-infected population, blood donors and pregnant women with hepatitis B surface antigen (HBsAg) prevalence rate of 1.02% – 10.6%.^8,15,16,17,18,19,20^ Amongst the blood donors in Botswana, the prevalence rate of HBsAg was 1.02%.^20^ Amongst the HIV-uninfected pregnant women, the prevalence was 1.1%.^19^ A recent study reported a 1.74% prevalence rate of HBsAg in HIV-infected mothers at the time of delivery, who were on treatment with zidovudine (ZDV) and lamivudine (3TC) or tenofovir (TDF) and emtricitabine (FTC) containing antiretroviral treatment ART regimens, which also have anti-HBV (antibody to hepatitis B virus) activity.^21^ Occult hepatitis B virus infection (OBI) was observed in 6.6% of HBsAg-negative women.^19^ There is paucity of data on the prevalence of viral hepatitis in patients with features of liver disease in Botswana. Studies done in 1985–1986 in the northern part of Botswana reported a high HBsAg seroprevalence rate of 13% – 47% and anti-HDV (antibody to hepatitis D virus) seroprevalence of 23% – 69%.^22^

Variation of anti-HDV seroprevalence from three out of four participants in one study to none of the nine participants were reported amongst women attending antenatal clinics in Botswana.^23^ Very low prevalence of HCV infection amongst blood donors (0.49%) and HIV-co-infected patients (0.8%) has been reported from Botswana.^17,18^

The aim of this study was to determine the serologic prevalence of viral hepatitis as a result of HBV, HDV and HCV and their risk factors amongst patients presenting with clinical features of hepatitis and/or altered liver function tests, attending Princess Marina Hospital (PMH), the main referral hospital in Botswana.

## Methods

### Study design and study population

Between February 2015 and July 2016, we conducted a cross-sectional study amongst outpatients and inpatients in the Department of Internal Medicine at PMH in Gaborone, Botswana. Princess Marina Hospital is the largest tertiary care, referral and teaching hospital in the country with about 530 inpatient beds. Patients are referred here from the primary and secondary care centres all over the country for specialist care. Eligible participants were those aged ≥ 18 years and with any of the clinical features of hepatitis including jaundice, history of liver disease and/or increased serum aspartate aminotransferase (AST), alanine aminotransferase (ALT), and serum bilirubin, of more than or equal to two times the upper limit of normal (ULN). The ULN, reported from the laboratory for ALT, AST and bilirubin were 41 U/L, 34 U/L and 25.7 mg/L, respectively.

### Sample size calculation

A sample size of 328 patients was estimated to show a 30% prevalence of HBsAg, with a two-sided type I error rate of 5% and 95% level of confidence.^24^

### Data collection

We collected patient information through an interviewer-administered questionnaire and reviews of patients’ charts and electronic medical records. The information included age, gender, history of jaundice or liver disease, hospital admissions, blood transfusion, haemodialysis, scarification, tattooing, vaccination for HBV, sexual behaviour, HIV status and intravenous drug use. After that, 5 mL of venous blood was collected by venepuncture, processed into serum and stored at –20°C for viral hepatitis serological testing.

### Laboratory methods

#### Hepatitis serological testing

Specimens were tested for respective viral hepatitis serological markers using commercially available enzyme-linked immunosorbent assay (ELISA), following manufacturer’s recommendations: HBsAg (*Diasorin Murex HBsAg Version 3, DiaSorin S.p.A. Dartford DA1 5LR, UK*); hepatitis B e antigen (HBeAg) (*DiaSorin ETI-EBK PLUS, DiaSorin, Saluggia, Vercelli, Italy*); antibody to hepatitis B core antigen (anti-HBc) (*Diasorin Murex anti-HBc (total) DiaSorin S.p.A. UK Branch)*; antibody to hepatitis B e antigen (anti-HBe) (*ETI-AB-EBK PLUS, DiaSorin, Saluggia, Vercelli, Italy*); antibody to hepatitis B surface antigen (anti-HBs), (*ETI-AB-AUK-3, DiaSorin, Saluggia, Vercelli, Italy*); anti-HDV (immunoglobulin M [IgM] and immunoglobulin G [IgG]) (*WANTAI HDV IgG and HDV IgM ELISA kit, Beijing Wantai Biological Pharmacy Enterprise, Beijing, China*) and antibody to hepatitis C virus (anti-HCV) were detected using MUREX ANTI-HCV (*VERSION 4.0) (DiaSorin S.p.A., Dartford DA1 5LR, UK*).

### Statistical analysis

Categorical variables were summarised using frequencies and percentages and continuous variables by median and interquartile ranges (IQR). Risk factors associated with hepatitis infection were analysed using univariate logistic regression. *P*-values < 0.05 were considered statistically significant. All analyses were performed using STATA version 13.1 (College Station, TX).

### Ethical considerations

Approval to conduct the study was obtained from the Office of Research and Development, University of Botswana (URB/IRB/1511), Botswana Ministry of Health (HPDME 13/18/1), and Princess Marina Hospital (PMH 5/79/173). All participants provided informed written consent before enrolment. Patients with positive results were referred to the infectious disease clinic for further management. No personal data have been included in this manuscript.

## Results

We enrolled 328 patients, of which 187 (57%) were females. Median age of the patients was 42.5 (IQR 30.9–53.1) years. A total of 317 (96.7%) study participants knew their HIV serostatus. Almost half of them (48.6%) were HIV-infected.

Any one of the serological markers of HBV infection was detected in 267 (81.4%) patients’ samples. HBsAg was detected in 153 patients (46.7%) of which 81 (53%) were females and 72% in the reproductive age group (18–49 years). Hepatitis B e antigen was detected in 26 (7.9%) samples, anti-HBc in 184 (56%), anti-HBe in 86 (26.2%), anti-HBs in 113 (34.5%) of the samples. Although, 175 (53.4%) samples were HBsAg negative, anti-HBs and/or anti-HBc were detected in 114 (65%) of these samples and 61 samples (35%) had no serological markers of HBV infection.

Amongst the HBsAg-positive females, 23 (28.4%) had anti-HBc and 14 (17.3%) had HBeAg. Serum aminotransferase were elevated in 19 (82.6%) of them. Risk factors for HBV infection were also analysed (Table 1).

**TABLE 1:** Risk factors in patients with positive **hepatitis B surface antigen.**

Risk factors	HBsAg + (*N* = 153)				HBsAg – (*N* = 175)				Unadjusted odds ratio	*p*
	Median	IQR	*N*	%	Median	IQR	*N*	%		
**Age in years**	40.3	30.0–54.3	-	-	39.8	32.0–51.4	-	-	0.9 (0.98–1.0)	0.700
Female	-	-	81	53.0	-	-	106	60.6	0.7 (0.47–1.14)	0.180
**Family history of jaundice** Yes	-	-	11	7.2	-	-	19	11.0	0.64 (0.29–1.38)	0.250
**Previous admission to hospital** Yes	-	-	62	40.5*	-	-	105	60.0	0.45 (0.29–0.71)	0.001*
**History of blood transfusion** Yes	-	-	29	19.0	-	-	38	21.7	0.8 (0.49–1.45)	0.540
**History of haemodialysis** Yes	-	-	3	2.0	-	-	1	0.6	3.5 (0.36–0.34)	0.250
**Recreational drug use** Yes	-	-	2	1.3	-	-	2	1.0	1.2 (0.16–8.2)	0.900
**Tattoos** Yes	-	-	14	9.2	-	-	12	7.0	1.4 (0.6–3)	0.440
**Traditional medicine ingestion** Yes	-	-	22	14.4	-	-	25	14.4	1 (0.54–1.9)	0.990
**Scarring by traditional healers or pastors** Yes	-	-	18	11.8	-	-	31	17.7	0.62 (0.33–1.2)	0.130
**Alcohol intake** Yes	-	-	33	21.6	-	-	24	13.7	1.73 (0.97–3.1)	0.060
**Smoking** Yes	-	-	16	10.5	-	-	12	7.0	1.59 (0.73–3.5)	0.240
**Sexually active** Yes	-	-	87	57.0	-	-	101	57.7	0.67 (0.62–1.5)	0.870
**Multiple partners** Yes	-	-	3	2.0	-	-	3	1.7	1.2 (0.23–5.7)	1.000
**Health care worker** Yes	-	-	4	2.6*	-	-	0	0.0	-	0.030*
**Liver malignancy** Yes	-	-	2	1.3	-	-	7	4.0	0.32 (0.07–1.6)	0.140
**History of sexually transmitted disease** Yes	-	-	20	13.0	-	-	26	14.9	0.86 (0.5–1.6)	0.640
History of vaccination	-	-	1	0.7	-	-	4	2.3	1.2 (0.07–2.12)	0.380
**HIV positive (*N* = 317)** Yes	-	-	64	42.7	-	-	89	53.3	0.65 (0.42–1.02)	0.060

* , *p*-value statistically significant.

HBsAg, hepatitis B surface antigen; IQR, interquartile ranges.

Amongst HBsAg-positive patients, associations were noticed in those with history of previous hospital admissions, and health care workers (HCW). On further analysis of the 105 HBsAg-negative patients with history of hospital admissions, it was observed that 67 (64%) were seropositive for ant-HBc, anti-HBe and anti-HBs and 38 (34%) were seronegative for any HBV marker.

Antibody to HDV was detected in 7 (4.6%) of the 153 HBsAg-positive samples. Median age was 46.7 years.

Antibody to HDV prevalence was slightly higher in males (57% vs. 43%, *p* = 0.58).

Antibody to HCV was detected in 14 (4.3%) out of 328 patients with 8 (57%) being females. Hepatitis B surface antigen was detected in five (35.7%) patients, and four (28.5%) were immune to HBV infection. Scarring by traditional healers or pastors was the only risk factor which was statistically significant as shown in Table 2.

**TABLE 2:** Risk factors in patients with positive antibody to hepatitis C virus.

Risk factors	Anti-HCV + (*N* = 14)				Anti-HCV – (*N* = 314)				Odds ratio (95% CI)	*p*
	Median	IQR	*N*	%	Median	IQR	*N*	%		
**Age in years**	46.7	33.8–56.3	-	-	39.4	31.0–52.4	-	-	1 (0.9–1.0)	0.200
**Gender** Female	-	-	8	57.0	-	-	179	57.0	0.65 (0.14–3.0)	0.900
**Previous admission to hospital** Yes	-	-	6	43.0	-	-	161	51.3	0.7 (0.2–2.0)	0.500
**History of blood transfusion** Yes	-	-	2	14.3	-	-	65	20.7	0.6 (0.14–2.9)	0.600
**Traditional medicine ingestion** Yes	-	-	2	14.3	-	-	45	14.4	0.99 (0.2–4.6)	0.900
**Scarring by traditional healers or pastors** Yes	-	-	5	35.7	-	-	44	14.0	3.4 (1.1–10.6)	0.026*
**Alcohol intake** Yes	-	-	4	28.6	-	-	53	17.0	2 (0.6–6.5)	0.300
**Sexually active** Yes	-	-	6	43.0	-	-	182	58.0	0.5 (018–1.6)	0.300
**History of sexually transmitted disease** Yes	-	-	1	7.0	-	-	45	14.3	0.46 (0.06–3.6)	0.500
**HIV infection** Yes	-	-	9	64.3	-	-	144	47.7	2 (0.6–6.0)	0.200

* , *p*-value statistically significant.

HCV, hepatitis C virus; IQR, interquartile ranges; CI, confidence intervals.

Sixty four (42.7%) HBsAg-positive patients were HIV infected. This includes two (3.16%) anti-HDV positive patients. Five (3.27%) of the HBsAg-positive patients were positive for anti-HCV and HIV co-infection also.

Nine (64.3%) of the 14 anti-HCV positive patients were also HIV infected.

## Discussion

In this hospital-based study amongst patients with liver disease, a high prevalence of seromarkers of viral hepatitis infections resulting from HBV, HDV and HCV were evident. In addition, higher prevalence of HBV and HIV, and HCV and HIV co-infections were observed.

The high prevalence of HBsAg (46.7%), indicating active or chronic infection, in this study is in consensus with that reported earlier from Botswana (47%) during an outbreak of non-A non-B hepatitis infection in 1985.^22^ This is higher than the seroprevalence reported in studies from patients with liver disease in India (25.9%), Mongolia (29.2%), Sudan (30%) and Ethiopia (35.8%).^9,10,12,25^ Studies amongst patients with chronic liver disease with smaller sample sizes have reported higher prevalence of 54.2% – 61.4% from Ghana and India.^13,26,27^ The presence of at least one serological marker of HBV infection in this study population (81.4%) agrees with reports from this region, where 70% – 95% of adults have serological evidence of past exposure to this infection.^15,22,24,28^ As this study enrolled patients with liver disease from the hospital, the prevalence reported here is higher than from studies conducted in other populations.^29,30^

Isolated presence of anti-HBc noticed in 6.3% patients, in the absence of HBsAg and anti-HBs, implies either recovering from acute HBV infection, distantly immune and test not sensitive enough to detect very low level of anti-HBs in serum, or susceptible with a false positive anti-HBc, or an undetectable level of HBsAg in the serum, and the person is actually chronically infected.^31^ In patients with isolated anti-HBc, screening for HBV DNA (deoxyribonucleic acid) is important to detect OBI which is defined as the presence of HBV DNA (< 200 IU/mL).^32^ Occult hepatitis B virus infection can develop after apparent and unapparent infection and maintains immunity by persistent antigen presentation which can favour the development of escape mutations.^32,33,34^ Patients infected with OBI can cause production of anti-HBc, which has been reported in 65.3% individuals with OBI.^33^ An early and effective immune response in cases of OBI can be responsible for the short life and low level HBs antigenemia.^34^ A proportion of patients considered to have cleared HBV infection (HBsAg negative and anti-HBc positive) may still harbour HBV cccDNA (covalently closed circular DNA) in their liver cells and are at risk of reactivation, especially when immunosuppressed.^35^ Transmission of HBV infection amongst OBI is through blood donations, organ transplantation, sexual contact, mother to child and to close contacts. It has also been associated with liver fibrosis, cirrhosis and hepatocellular cancers.^33,36^ Prevalence of OBI varies from 1% to more than 15% amongst HIV-positive patients in the USA and South Africa. In Botswana, the prevalence of OBI was 26.5% amongst the HIV-positive treatment naïve patients which decreased to 1.5% after initiation of highly active antiretroviral therapy (HAART).^33^ In the study amongst pregnant women in Botswana, a 6% prevalence of OBI was reported, 7.4% in HIV-positive women and 5.7% in HIV-negative women.^19^

The coexistence of HBsAg and anti-HBs (18%) detected in this study has been reported in 5% to 30% of HBsAg-positive individuals and they are considered as carriers of HBV.^37^ This has been attributed to the antibodies being unable to neutralise the circulating virions or to the emergence of escape mutants during the natural course of the HBV infection.^37,38,39^ This coexistence has also been associated with increased HBsAg seroclearance and hepatocellular carcinomas.^40^ Hepatitis B e antigen detected in 7.9% patients indicates higher levels of replication and higher infectivity. Patients may be in the phase of acute or chronic active HBV infection.

Isolated HBeAg, without HBsAg or anti-HBc, was detected in three of the samples with very high levels of ALT and AST (> 10 times the ULN). This may be because of HBsAg mutant or low undetectable levels of HBsAg with the presence of HBV DNA.^41^ High titres of liver enzymes imply inflammation and liver damage primarily caused by cytotoxic T lymphocyte-mediated reactivity against infected hepatic cells presenting major histocompatibility complex (MHC) class I bound T cell peptide epitopes.^42^

In Africa, HBsAg seroprevalence in males relative to females ranges from 1.13:1 to 3:1 usually attributed to the pattern of male sexual activity.^43^ Similar to other studies from Botswana amongst HIV-infected cohort, this study also noted a slightly higher prevalence amongst the females (1:1.12%), which is not statistically significant.^17,44^ In this study, 72% of HBsAg-positive females were in the reproductive age group with positive anti-HBc in 28.4% and 60% of them were HBeAg positive indicating high infectivity and can be in the acute hepatitis or chronic active hepatitis stage as a result of recent infection or reactivation.

In this study, a higher number of hospital admissions were reported by the HBsAg-negative patients and a statistically significant association was noted amongst HBsAg positive and negative patients with history of hospital admission. On analysis, it was observed that 64% of the HBsAg-negative patients at the time of testing had HBV infection in the past, with serological evidence of anti-HBc and anti-HBs in their sera and only 36% of them were never exposed to HBV. This must have contributed to the increased number of hospitalisation amongst this group.

Interestingly, all the four HCW in this study who tested positive for HBsAg, were in the chronic infectious stage with positive anti-HBc and anti-HBe in their sera and negative anti-HBs. A study carried out amongst HCW in South Africa found that only 30.6% were immune to HBV infection with presence of anti-HBs in their sera.^24^ A similar rate (31%) has also been reported from Botswana.^45^ This implies that all HCW should be screened and vaccinated against HBV infection.^46^

It is to be noted that five patients in our study self-reported to have received vaccination against HBV and only three had anti-HBs in their sera. One of them had active infection with positive HBsAg, HBeAg and anti-HBc and negative anti-HBs indicating unreliability in self-reporting. Antibody to hepatitis B surface antigen alone was detected in 6.7% of the patients indicating remote infection or previous vaccination.^39,47,48^ Vaccination for HBV infection was introduced in Botswana in 1995, but none of the 12 participants below 20 years reported having received it.^49^

Botswana reported a 95% national HBV vaccine coverage by 2011, but its timely administration coverage (birth dose) was 74%.^50^ In a recent study to investigate HBV vaccine responses in HIV-exposed but uninfected children in Botswana, a 98.9% protective immunity was reported in this cohort by 18 months of age and only 75% had received the timely birth dose.^21^ Decline in immunity from vaccination has been reported from Malawi and South Africa in adolescence leading to an increased risk of acquisition of HBV infection because of sexual activity or injecting drug use.^2,51^ Vaccine failures also have been reported as a result of development of escape mutants.^51^

The anti-HDV prevalence (4.6%) amongst HBsAg-positive patients was lower than that reported in 1985–1986 from the northern part of Botswana where it had a higher (23% – 69%) prevalence in the population at that time.^3,22^

In patients with liver disease in Africa, anti-HDV prevalence rates of 1% – 66.7% have been reported.^3,14^

The anti-HCV seroprevalence in this study was higher (4.3%) than the previously reported rates of 0.49% – 0.8% in HIV-infected patients in Botswana, likely because of the fact that the enrolled patients had liver disease.^17,18,52^

Higher anti-HCV seroprevalence between 6% and 22.5% has been reported from similar studies in Africa and Asia.^5,10,12,26^ Similar to other studies in this region a slightly higher prevalence (57%) was found in females.^53,54,55^ As reported in previous studies, scarring by traditional healers or pastors was associated with anti-HCV positivity.^18^ This might be because of unsterile practices during the procedures. In our study, there were no intravenous drug users or men having sex with men in whom usually the prevalence is high.^18,56,58^

The estimated prevalence of HBV/HCV dual infection worldwide is approximately 5% – 20% in HBsAg-positive patients and 2% – 10% in HCV–positive patients because of the similar route of transmission.^57,59^ In this study, 35.7% anti-HCV positive patients were HBsAg positive indicating active or chronic HBV infection.

HIV co-infection rates of 4.1% – 28.4% have been reported in individuals with chronic HBV infection in Africa and may be because of common routes of transmission.^57,59^ In our study, there was a much higher prevalence of HIV co-infection (42.8%) which may be because of the higher prevalence of HIV infection (20.3%, UNAIDS 2019) in the population studied.^42^

HIV co-infection was higher (68%) also amongst anti-HCV positive patients in this study, perhaps resulting from the persistence of HCV viremia in HIV-infected patients.^56^

Patients negative for any serological markers of hepatitis (18.6%) had other comorbid conditions such as tuberculosis infection, or were on treatment for diabetes, hypertension, tuberculosis, HIV or had history of alcohol abuse. These conditions may have contributed to their hepatic injury.

## Limitation

The study population was a convenience sampling from hospital inpatients and outpatients with signs and symptoms of liver disease and so was not representative of the people of Botswana.

As PMH is a referral hospital, we managed to enrol patients from all the 10 districts in Botswana though not in equal proportions. Samples were collected only once, so the progression of illness could not be assessed.

The results may be an over-reporting of the prevalence of viral hepatitis infection because the study was hospital-based. Further research and surveillance in the community and in different centres across the nation is warranted. The data on risk factors were self-reported by the patients, so certain facts may not be reliable, for instance HIV status, sexual history, number of partners and drug use. As a result of financial constraints, anti-HBc IgM, HBV DNA and HCV RNA (ribonucleic acid) were not determined in this study. This would have helped in better classifying the patients and establishing particularly whether they had acute or chronic HBV or HCV infection. Therefore, acute hepatitis, OBI and confirmation of HBV and HCV infections were not detected.

## Conclusion

This study reports a high seroprevalence of HBV, HCV, HDV infection markers and HBV/HIV co-infection in a population with liver disease observed in the main referral hospital in Botswana. A routine hepatitis profile screening of all patients with clinical manifestations of liver disease is therefore recommended as the seroprevalence of markers of viral hepatitis is high amongst this group along with test for HBV DNA to detect OBI and in turn prevent transmission. A nationwide surveillance study for HBV infection and vaccine response will inform the policymakers regarding impact of the prevention activities. Health care workers are at particular risk of HBV infection, so immunisation is highly advisable. Periodical epidemiological data collection on the prevalence of different agents causing viral hepatitis is required to guide the policymakers to make decisions regarding prevention, diagnosis and management of viral hepatitis and also to evaluate the impact of disease prevention and control activities. Identification and treatment of individuals infected with hepatitis at the earliest will help the country to reach the WHO target of elimination of hepatitis by 2030.
